# A Genomic and Transcriptomic Analysis of the C-Type Lectin Gene Family Reveals Highly Expanded and Diversified Repertoires in Bivalves

**DOI:** 10.3390/md21040254

**Published:** 2023-04-20

**Authors:** Amaro Saco, Hugo Suárez, Beatriz Novoa, Antonio Figueras

**Affiliations:** Institute of Marine Research IIM-CSIC, 36208 Vigo, Spainbeatriznovoa@iim.csic.es (B.N.)

**Keywords:** C-type lectins, lectins, bivalves, innate immunity, evolution, component system, Mollusca

## Abstract

C-type lectins belong to a widely conserved family of lectins characterized in Metazoa. They show important functional diversity and immune implications, mainly as pathogen recognition receptors. In this work, C-type lectin-like proteins (CTLs) of a set of metazoan species were analyzed, revealing an important expansion in bivalve mollusks, which contrasted with the reduced repertoires of other mollusks, such as cephalopods. Orthology relationships demonstrated that these expanded repertoires consisted of CTL subfamilies conserved within Mollusca or Bivalvia and of lineage-specific subfamilies with orthology only between closely related species. Transcriptomic analyses revealed the importance of the bivalve subfamilies in mucosal immunity, as they were mainly expressed in the digestive gland and gills and modulated with specific stimuli. CTL domain-containing proteins that had additional domains (CTLDcps) were also studied, revealing interesting gene families with different conservation degrees of the CTL domain across orthologs from different taxa. Unique bivalve CTLDcps with specific domain architectures were revealed, corresponding to uncharacterized bivalve proteins with putative immune function according to their transcriptomic modulation, which could constitute interesting targets for functional characterization.

## 1. Introduction

The innate immune system, present both in vertebrates and invertebrates [1], is able to discriminate between self and nonself and to generate a rapid response to infections. This response is mediated by molecules known as pattern-recognition receptors (PRRs), which recognize microbial molecules known as pathogen-associated molecular patterns (PAMPs), such as lipoproteins, bacterial or viral nucleic acids, and carbohydrates [2].

Lectins are a well-characterized family of PRRs capable of reversibly binding to carbohydrates and glycoconjugates [3,4]. These proteins are conserved through evolution, present in viruses, bacteria, plants, fungi, invertebrates and vertebrates, and play key roles in several processes involving self and nonself recognition [5,6]. Because of their ubiquitous, abundant, and diverse nature, lectin classification can follow different criteria [7,8]. They can be studied according to the carbohydrate for which they show the highest specificity: glucose/mannose, galactose/N-acetylgalactosamine, N-acetylglucosamine, fucose, and N-acetylneuraminic acid [9]. The other classification system is based on defining features such as their structure and domains [10,11,12]. C-type lectin-like proteins (CTLs) are a superfamily of lectins characterized by a domain conserved across all Metazoa [13]. These lectins are crucial in the innate immunity of invertebrates and vertebrates, as they function as pattern recognition receptors, binding carbohydrates on the cell surfaces of pathogens through their Ca^2+^-dependent carbohydrate recognition domains (CRDs) [14,15]. CTLs play key roles in the complement system, activating the central component “C3” and binding to the surface of pathogens, helping to eliminate them by their binding to specific complement receptors on immune cells, lysis, or opsonization [16]. In addition, CTLs are also involved in tissue homeostasis and clearance of apoptotic and necrotic cells [17,18]. CTLs are composed of lectins with different specificity targets, such as galactose, mannose, or fucose, although their classification is usually based on their domain organization, which is the criteria followed for the different groups defined in vertebrates [19]. Metazoans usually have very large CTL gene families, in contrast to the very low numbers found in plants and microbes [7,19]. These proteins have undergone lineage-specific expansions throughout the evolution of different metazoan phyla [19,20,21], causing a usual lack of homology between distant species, with each phylum developing its own CTL repertoires.

Mollusks are a large phylum of invertebrate animals present in terrestrial, freshwater, and marine environments throughout the world. As invertebrates, they depend solely on an innate immune system, which has been proven to be highly efficient in these species. The Bivalvia class is constituted mainly of sessile marine filter feeders that constantly filter the surrounding water, incorporating nutrients and pathogens. These species are characterized by immune gene families that have large lineage-specific expansions and presence/absence variation [22,23,24]. Diversity in these gene families is driven by functional diversification to adapt their innate immune system to the wide range of pathogens present in the marine medium [25,26]. Expansions have been described in complement-related immune proteins implicated in pathogen binding, such as C1q proteins [27,28] and fibrinogen-related proteins [29,30]. In lectins, there are also indications that point to an expansion [31], although the phenomenon has not yet been studied in detail. CTLs have been described in these species, presenting the conserved domain structure, although calcium dependency does not happen in all cases [32,33]. It has been described that CTLs present greater variability in mollusks than in vertebrates; for example, in mollusks, there are more than 10 variants in one particular site of the CRD domain, which remains unchanged in vertebrates [34]. Based on these indications, the variability and possible expansion of CTLs in these species was the study of this work.

As in metazoans, molluscan lectins have also been associated with self and nonself recognition, reproduction, tissue adhesion, and innate immunity [35]. C-type lectins in particular play a fundamental role in bivalve immunology, taking part in different processes such as PAMP recognition and binding [36], agglutination of various microbes [37], induction of phagocytosis [38], and even antibacterial activity [39]. CTLs also display nonimmune functions in mollusks, such as in the recognition of food particles in bivalves [40,41]. Because of this functional diversification, mollusk lectins have been explored in the biomedical and biotechnological industries. The great biodiversity found in marine organisms provides great potential targets for the identification, purification, and discovery of new drugs [35]. Several mollusk lectins have been functionally characterized, exhibiting antifungal [42,43], bacterial-binding, and antibacterial [44,45,46] or biomineralization [47] properties. More advanced biotechnological applications have used mollusk lectins for cancer diagnosis and treatment because of their specific binding and apoptosis-induction activity [48,49]. MytiLec-1 is a lectin from the bivalve mollusk *Mytilus galloprovincialis* with implications in cell death induction and bacteriostatic activity [50,51], and it has been used as a model for the design of an artificial lectin protein for novel cancer treatments and diagnostics [52].

Although CTLs have been described as a generally diverse and expanded gene family in metazoans, in previous evolutionary studies, mollusks were an underrepresented group [19,53]. The objectives of this work were to study and increase the knowledge on the diversity and evolution of C-type lectin-like domain-containing proteins in mollusks, with a special focus on bivalve species, and to study the overall metazoan diversity as context. This work aimed to characterize for the first time the CTL expansion that occurs in bivalve species so that specific subfamilies within this great diversity could be revealed, allowing the creation of classified repertoires. Phylogenetic and orthology analyses were employed to study CTLs in different species, which revealed the weight of intraphylum conservation and the degree of lineage-specific expansions in the origin of their repertoires. Expression analyses were conducted with the CTLs of a specific bivalve species using a large transcriptomic dataset that included experiments conducted with several stimuli, with the objective of identifying lectins with particular functional roles that could be targets for future functional characterization studies.

## 2. Results

### 2.1. Distribution of C-Type Lectin-like Proteins in Metazoa

**Figure 1:**
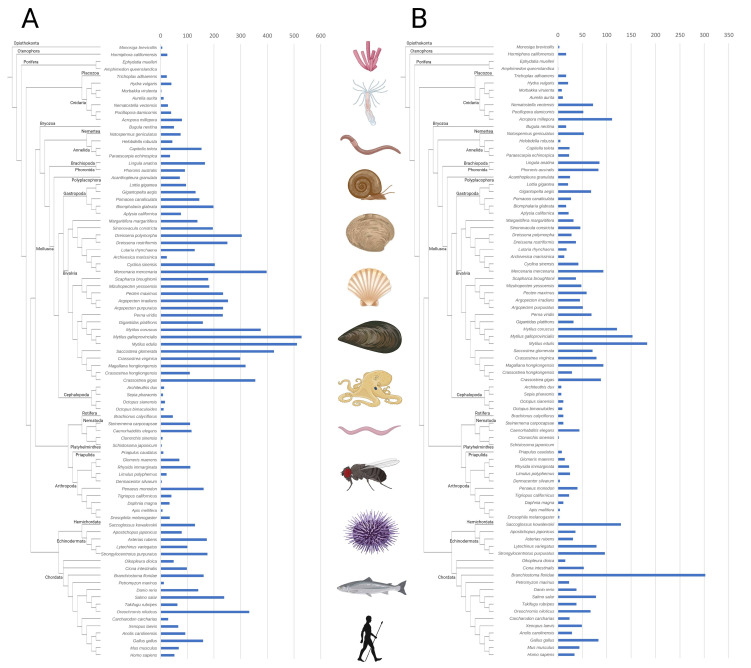
Distribution of C-type lectin-like proteins across the analyzed metazoan species. (**A**): Number of genes encoding proteins with only C-type lectin-like domains (CTLs). (**B**): Number of genes encoding proteins containing C-type lectin-like domains in addition to other domains (CTLDcps).

The C-type lectin-like repertoire was studied in the genomes of different representative metazoan species, and two protein types could be identified: (a) proteins containing only C-type lectin-like domains (designated CTLs), whose distribution is shown in Figure 1A, and (b) proteins containing other domains in addition to the C-type lectin-like (designated CTL domain-containing proteins, or CTLDcps), shown in Figure 1B. Regarding the CTLs, their presence was detected in all metazoan species. Only one CTL protein was detected in each poriferan species, and generally small numbers of CTL genes were found in other ancient taxa, such as choanoflagellates, platyhelminths, priapulids, and most cnidarians. Regarding deuterostomes, while humans and other chordates presented gene families with approximately 50 CTL genes, expanded CTL repertories were found in some echinoderms and chordates, such as lancelets (*Branchiostoma floridae)* and avians (*Gallus gallus*), and especially in teleost fishes, such as *Salmo salar* and *Oreochromis_niloticus*, with over 200 and 300 CTL genes. Large variations between the repertoires of species from the same phyla were observed in Arthropoda and Annelida, and large families with approximately 100 CTLs were found in the analyzed nematods, brachiopods, and phoronids. Regarding mollusks, cephalopods had a very small number of CTLs, while gastropods had more than 100 CTLs for most cases, and bivalves had the largest repertoires, with approximately 200 CTLs and up to 300–500 CTLs in many species, especially mussels and oysters. With respect to CTLDcps, the repertoires were generally smaller, below 50 genes for most species and reaching the largest numbers in some bivalves, some cnidarians, chordates, and other less-represented phyla. A striking case was the chordate lancelet (*Branchiostoma floridae)*, with 300 CTLDcp genes.

### 2.2. Orthology and Phylogenetic Analyses of CTL Gene Families

**Figure 2:**
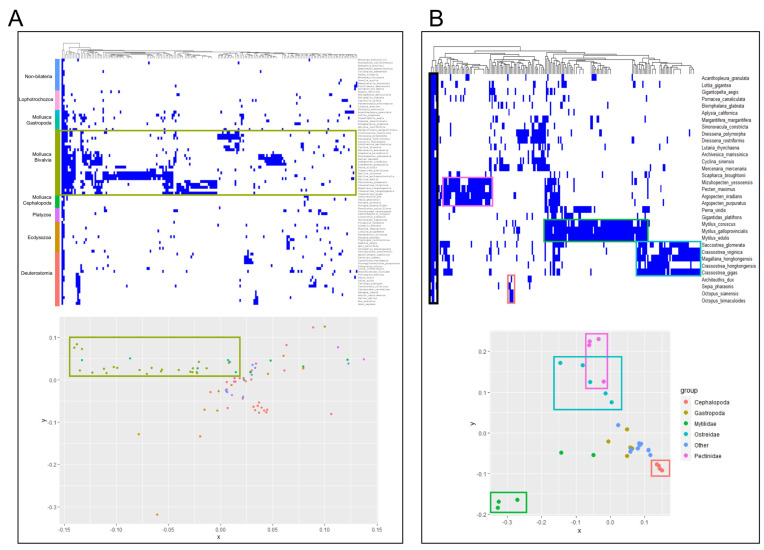
Orthology analyses of genes encoding only C-type lectin-like domains (CTLs). (**A**): Orthology analysis of CTLs across the analyzed metazoan species. The heatmap displays all CTL orthology groups and the degree of conservation between all species for each one. The PCA distributes all species according to the similarity of their CTL repertoires based on the orthology relationships displayed above. The color legend is indicated in the heatmap. Certain fragmentation could be observed, indicating lineage-specific repertoires with orthology relationships only between close species, particularly in bivalves (highlighted). (**B**): The same results are shown for a phylum-level orthology analysis performed only with mollusk species. General conservation across all mollusks is highlighted in black, while clear clusterization is shown for some groups of species, particularly *Mytilidae*, *Ostreidae*, and *Pectinidae* bivalves and cephalopods, indicating conserved linage-specific CTL repertoires.

Once all the CTLs shown in Figure 1A were identified, their orthology relationships were retrieved from an orthology analysis performed with all the analyzed metazoan species (Figure 2A). The heatmap clearly showed fragmented orthology results, indicating that each lineage of species had its own CTL gene family without clear orthologs in other phyla. However, the presence of some clusters in the heatmap indicated orthology conservation between CTLs of close species, especially in bivalves, which were overrepresented in the analysis. The PCA distribution showed low CTL conservation between different species and different phyla. However, as in the heatmap, the slight grouping observed in bivalves indicated that this clade could have a certain common repertoire. Therefore, the analysis was performed again but using only mollusk species to gain more resolution (Figure 2B). Even if some degree of fragmentation in the analysis was still clear, a cluster of orthology groups with different degrees of common conservation among mollusks was detected (and highlighted by a black square in the heatmap). Furthermore, clear orthology between close species was detected and indicated in the heatmap and in the PCA, namely, in mussels from the Mytilidae family (*M. galloprovincialis*, *M. edulis, M. coruscus*) (shown in green), in oysters from the Ostreidae family (*Saccostrea glomerata, Crassostrea virginica*, *Magallana hongkongensis*, *C. hongkongensis*, *C. gigas*) (shown in light blue), in scallops from the Pectinidae family (*Argopecten irradians*, *A. purpuratus*, *Mizuhopecten yessoensis*, *Pecten maximus*) (shown in pink), and in the small repertoires of cephalopods (*Octopus bimaculoides*, *O. sianensis*, *Sepia pharaonis*, *Achiteuthis dux*) (shown in orange).

**Figure 3:**
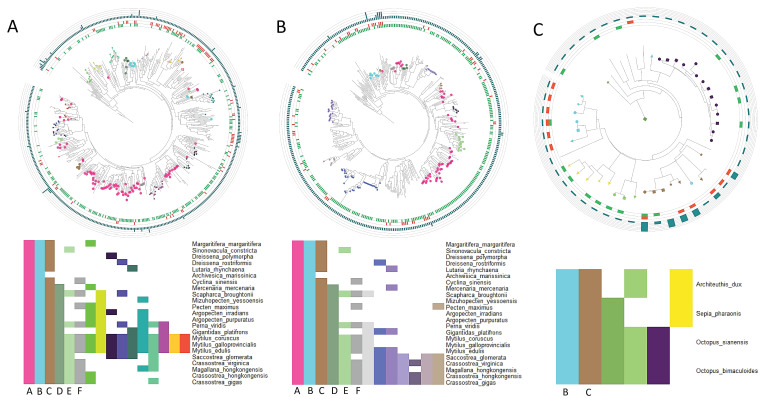
C-type lectin-like (CTL) subfamilies and their conservation across mollusks in three lineage-specific repertoires. (**A**): Phylogenetic analysis of C-type lectin-like proteins (CTLs) in *Mytilus galloprovincialis* and CTL subfamilies shared by *Mytilidae* mussel species. CTL orthology groups shared by the three *Mytilidae* species are shown, along with their conservation degree at the class level among bivalves. In the phylogenetic tree, these CTL orthology subfamilies are indicated by each color. The presence of a single peptide (green bars), the presence of a transmembrane domain (orange bars), and the number of CTL domains (outer cyan bars) are indicated for each CTL protein in the phylogenetic tree. (**B**): Phylogenetic analysis of CTL proteins in *Crassostrea gigas* and CTL subfamilies shared by all *Ostreidae* oyster species. Distribution in the phylogenetic tree and orthology conservation of such subfamilies between bivalves is indicated. The presence of a single peptide, the presence of transmembrane domains, and the number of CTL domains are also shown. (**C**): Phylogenetic analysis of CTL proteins encoded in all the analyzed cephalopod species and orthology conservation of such CTLs. The presence of a single peptide, the presences of transmembrane domains, and the number of CTL domains are also shown. By comparing the orthology conservation of each lineage-specific repertoire, two orthology subfamilies conserved at the mollusk level could be identified (B, C), as well as an additional subfamily conserved between all bivalves (A). Additionally, other subfamilies were shared between the two bivalve lineage-specific repertoires of mussels and oysters (D, E, F).

Phylogenetic analyses were performed to further characterize these molluscan conserved repertoires using *M. galloprovincialis* as a representative of mussels (Figure 3A), *Crassostrea gigas* CTLs as a representative of oysters (Figure 3B), and cephalopod CTLs (Figure 3C). The structure of these CTLs was retrieved and indicated in the trees (presence of signal peptide or transmembrane domains and number of CTL domains). The main CTL subfamilies present in each group of species were annotated in each tree. The orthology conservation of these subfamilies with other species was also represented in additional heatmaps. Two CTL subfamilies (designated B and C) were conserved at the phylum level in mollusks, with orthologs in all bivalve and cephalopod species. Another CTL subfamily (designated A) was conserved in all bivalves but absent in cephalopods. This subfamily consisted of small secretable lectins and was quite expanded in both mussels and oysters (pink dots in the trees of Figure 3A,B). Three other CTL subfamilies (D, E, F) were present in both mussels and oysters, and their orthology study revealed partial conservation with other species of the Bivalvia class. The other subfamilies were specific to each lineage and conserved at the family level (present in mussel species or in the oyster species), while the detection of orthologs in other bivalves was rare.

### 2.3. C-Type Lectin-like Expression Data

**Figure 4:**
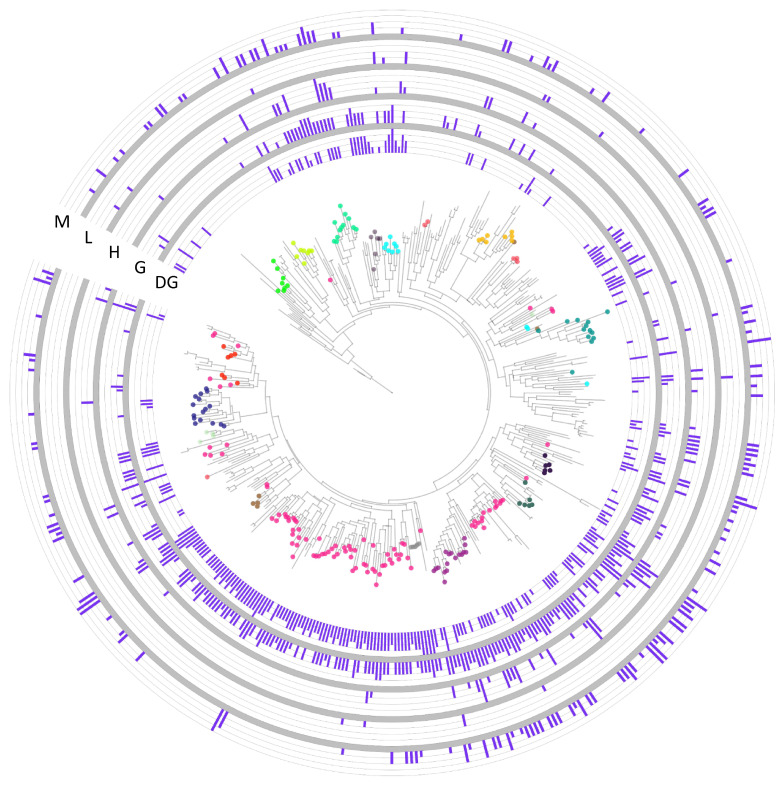
Expression of the mussel (*Mytilus galloprovincialis*) CTL gene family. The same phylogenetic analysis of *Mytilus galloprovincialis* C-type lectin-like proteins (CTLs) shown in Figure 3A is included here, along with the expression level of each CTL protein. The maximum level of expression that each protein reached in the digestive gland (DG), gills (G), hemocytes (H), mid-trochophore larvae (L), and mantle (M) across the analyzed SRA transcriptomic datasets (Supplementary File S2) is shown. Colors in the phylogenetic analysis show the orthology subfamilies identified in Figure 3. For example, subfamily “A”, conserved at the Bivalvia level, was mainly expressed in digestive glands and gills with almost no expression outside of mucosal tissues.

To further infer the possible function of the expanded CTLs in bivalves, we retrieved expression data from the mussel *Mytilus galloprovincialis* and compared the pattern of CTL expression in different sample types (Figure 4). Interestingly, phylogenetic branches corresponding to CTLs from subfamily A, which was conserved at the Bivalvia level, showed high gene expression in the digestive gland and gill but not in other tissues. Only a few CTLs showed higher expression levels in hemocytes than in mucosal tissues, such as gills or digestive glands, which could indicate the particular importance of CTLs in mucosal immunity in mussels.

**Figure 5:**
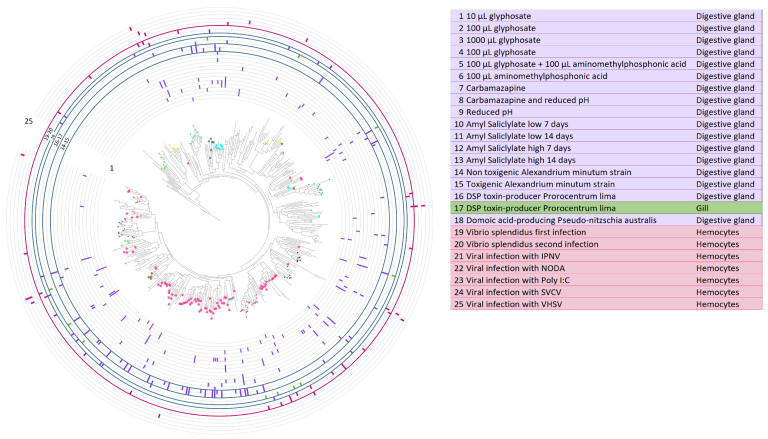
Modulation of specific mussel CTL genes with different transcriptomic stimuli. The phylogenetic tree of *Mytilus galloprovincialis* CTLs is included along stacked histograms indicating the modulation (*p* value < 0.05) of specific CTLs with different transcriptomic stimuli (Supplementary File S3).

Lectin gene modulation was also studied in transcriptomes under different stimuli and/or biotic or abiotic stress (Figure 5). CTLs from subfamily A, which were specifically expressed in the digestive gland and gills, were modulated with several toxic stimulations in the digestive gland with the toxin-producing species *Alexandrium minutum* and *Pseudonitzschia australis*. Stimulations with chemicals or contaminants also modulated CTLs in the digestive gland. Several modulated lectins were also detected in hemocytes, especially after stimulation with pathogenic bacteria, although viral stimulation also induced certain regulation of expression.

### 2.4. CTL Domain-Containing Proteins (CTLDcps)

**Figure 6:**
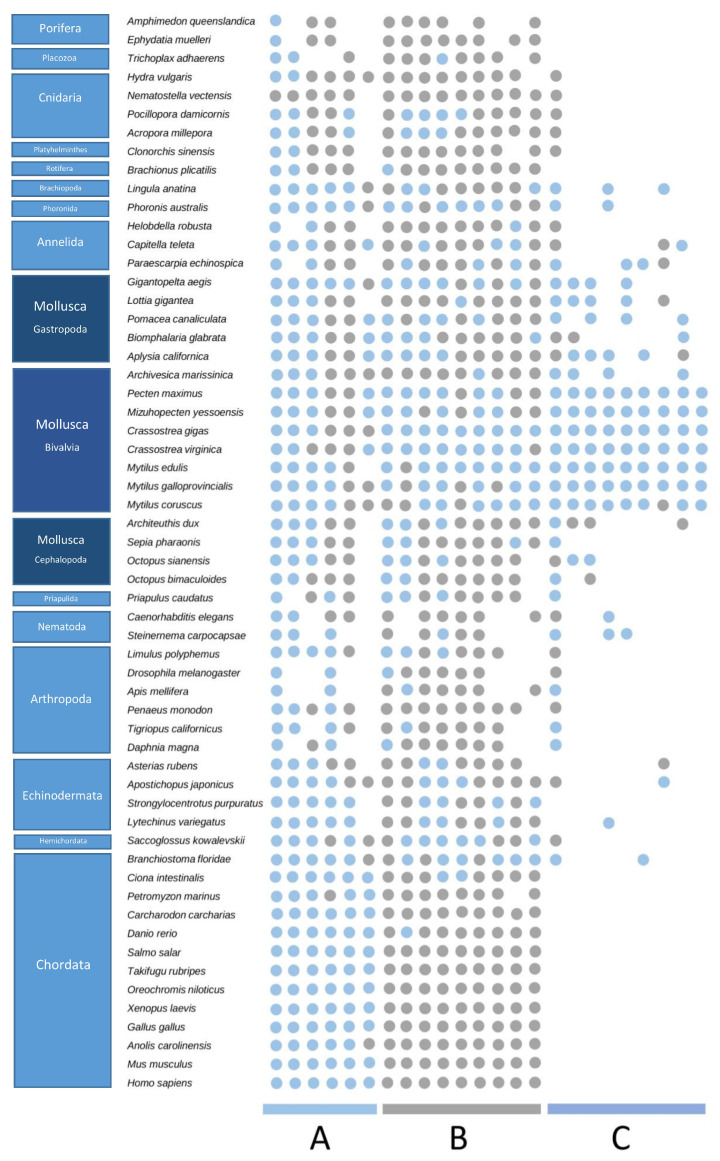
Orthology analysis of proteins containing C-type lectin-like domains in addition to other domains (CTLDcps). For a subset of the analyzed metazoan species, the conservation of several CTLDcps orthology groups is indicated: blue: orthologs including the CTL domain; gray: orthologs without the CTL domain. Three large categories were revealed: (A) orthology groups/gene families containing CTL domains across all metazoans and conserved in vertebrates; (B) gene families containing CTL domains in some metazoans (especially bivalves) while the CTL domains were lost from the ortholog genes of other species (completely lost in vertebrates); (C) CTLDcps gene families unique in bivalves, without clear orthology in other metazoan clades.

The set of proteins that contained C-type lectin-like domains in combination with different additional domains was also studied in the metazoan genomes (Figure 1B). As in the case of CTLs, an orthology analysis was used to study the metazoan CTLDcps. In Figure 6, several orthogroups are represented for a subset of those species. In this figure, each column of data corresponds to an orthogroup containing CTLDcps, and for each species, it is indicated whether their orthologous proteins have the C-type lectin-like domain or not. Three main types of orthogroups were identified: (A) proteins that conserved the same domain architecture, with the CTL domain generally found in all species; (B) proteins that were only present in the CTL domain in bivalves, some deuterostomes and other invertebrates, while the CTL was absent in orthologs from most species, especially vertebrates; and (C) proteins that did not show general orthology outside bivalves, which therefore constituted bivalve-specific CTLDcps. The most extreme changes were observed in chordates: all species showed the CTL domain in orthologous proteins from group “A”, while the CTL domain was absent from all chordates in the “B” orthology groups, and they did not show any orthology with bivalve proteins from group “C”.

**Figure 7:**
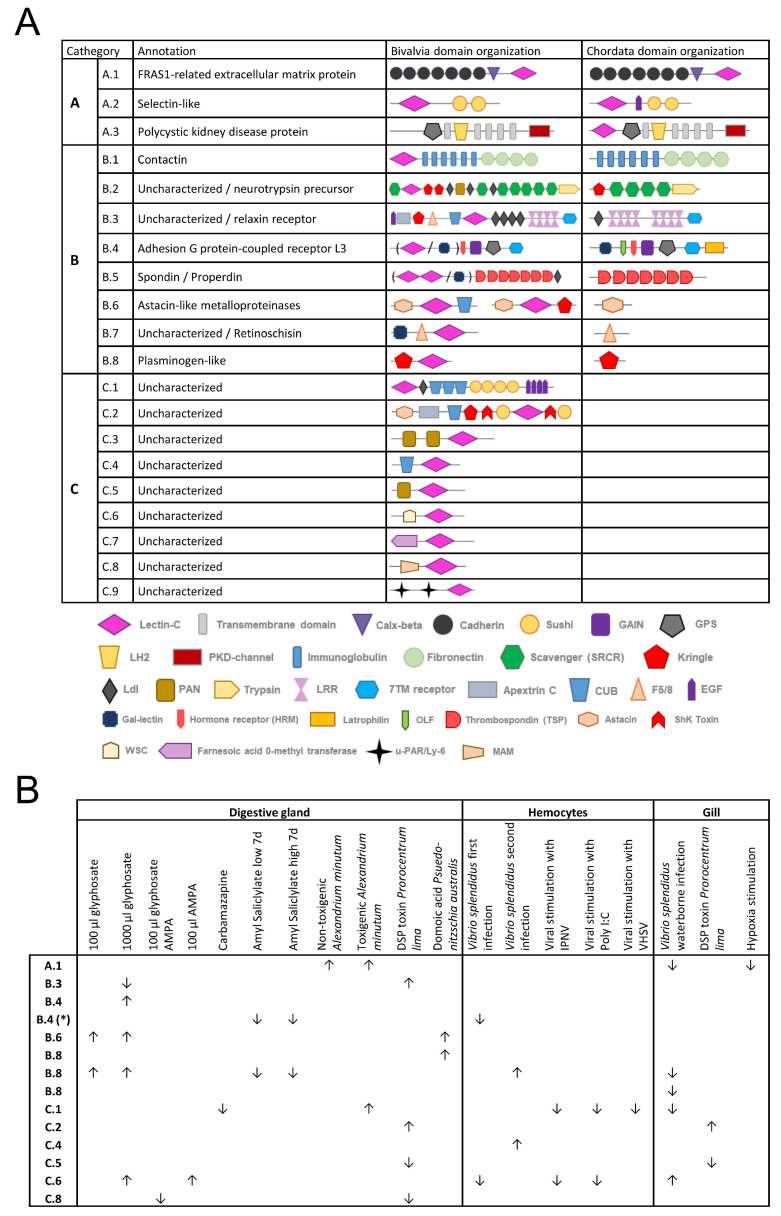
Domain architecture and transcriptomic modulation of some C-type lectin-like domain-containing protein (CTLDcp) bivalve gene families. (**A**): To exemplify the obtained results, several gene families were chosen as examples for the three categories of CTLDcps presented in Figure 6. The annotation of each gene family is indicated, along with the domain architecture found in mussels (as an example of bivalves) and in human orthologs (as an example of vertebrates). The general conservation of CTL domains in both orthologs could be observed in category “A” CTLDcps, while the lectin domain was lost from the vertebrate orthologs of category “B”. CTLDcps from categories “B” and “C” included unique domain architectures found in bivalves and corresponded mainly to uncharacterized proteins that could be of interest for future research. (**B**): The transcriptomic modulation (*p* value < 0.05) found for the mussel CTLDcps collected in Panel A is shown. Samples corresponding to each transcriptomic experiment are indicated in Supplementary File S3. *, Gal-lectin domain instead of C-type lectin domain; ↑, up-modulated expression; ↓, down-modulated expression.

Therefore, two reference species were selected, *M. galloprovincialis* for bivalves and *Homo sapiens* for chordates, and used in Figure 7A to exemplify specific proteins from the three categories. It can be observed how category “A” proteins could share the same domain structure between orthologs from chordates and bivalves, as occurred with the “FRAS1-related extracellular matrix protein” orthologs (A.1), or could even lose the CTL in bivalves while conserving it in chordates, as with the “Polycystic kidney disease protein” orthologs (A.3). “B” proteins behaved in the opposite manner. Orthologous proteins with similar domain architectures did not show the CTL domain in chordates, while it was found in bivalves. Some of these proteins were unannotated or unknown in bivalves, while others were known for their chordate ortholog. Finally, category “C” included CTLDcps specific to bivalves, all of which are uncharacterized. Modulation was observed for some of these bivalve CTLDcps in the mussel expression dataset (Figure 7B), including the uncharacterized mussel proteins of the “C” category, with C.2 and C.5 following the same modulation after DSP toxin stimulation in the gill and digestive gland or with C.1 upregulated only with *Alexandrium minutum* toxin stimulation.

## 3. Discussion

CTLs are ubiquitously found in the tree of life and are present even in bacteria, although with different domain structures [19]. Among eukaryotes, CTLs are scarce in plants and, as our results showed, highly abundant and globally distributed in metazoans, demonstrating that massive expansions took place in the metazoan lineages [7,53]. We observed consistent patterns among the studied animal clades. The few CTL genes found in Porifera are in agreement with previous findings [53], and they were shown to act as aggregation factors [54,55]. These reduced repertoires may have been caused by gene loss, since larger repertoires were found in the choanoflagellate metazoan ancestor *Monosiga brevicollis* and in other ancestral metazoans such as ctenophores, placozoans, and cnidarians. In bilaterians, CTL repertoires were generally larger both among protostomes and deuterostomes. Lineage-specific expansions and specializations drove CTL evolution, as reflected in the changing size of the gene family, the lack of orthology, and the variable domain architectures found among the CTLDcps of different lineages [19,56]. This evolutionary history shows evidence of its being directly related to functional diversification needs. The specificity of innate immunity has been demonstrated to be generated and maintained by the expanded CTL repertoires of several invertebrates, such as insects, crustaceans, and nematodes [53]. In particular, nematodes represent a good example of the relationship between the CTL gene family and the functional needs of the lifestyle of each species, as the size and domain diversity of CTL repertoires in insect-vectored nematodes was dramatically reduced compared to free-living species [57].

The analyzed set of species was enriched in mollusks, which allowed us to reveal the highest levels of expansion of C-type lectin-like proteins in this group when compared with all the remaining species. Bivalves in particular showed the most general and largest expansion, with more than 200 genes for most species, with sometimes more than 400 or even 500 genes. Only one bivalve had fewer than 100 CTL genes, *Archivesica marissinica*, which could be due to a massive loss or most likely to a genomic artifact. The largest repertoire was retrieved from the mussel *Mytilus galloprovincialis*, which was chosen to perform expression analyses in search of evidence of CTL functional specialization. The bivalve expansion contrasts with the strikingly reduced repertoires of the Cephalopoda class. Significant differences in the expansion magnitude of different gene families, including CTLs, have been observed between bivalves and other mollusks [24,31]. Bivalves are characterized by the general expansion of several immune gene families, gaining an immunological specificity that is needed to address the great diversity of the potential marine pathogens that they face during their constant filtration of sea water [24,27,58,59]. Bivalve genomes possess high levels of hemizygosity that act as reservoirs of genetic diversity, increasing the number and diversity of genes at the population level [22]. The fact that these hemizygous regions are enriched in immune genes is one of the mechanisms that can drive the important diversification in these gene families in bivalves [23]. Despite the common general expansion, bivalves present high levels of CTL diversity among themselves, with extensive orthology conservation occurring only inside specific lineages of close species such as mussels (*Mytilidae*), oysters (*Ostreidae*), or scallops (*Pectinidae*). Only three CTL subfamilies showed orthology among all bivalve species (with the *Archivesica* exception mentioned before), and two of those (B and C) were conserved at the Mollusca level, being found in the reduced repertoires of cephalopods as well. Therefore, subfamilies B and C would be the most ancestral mollusk CTL subfamilies, from which the other diverse forms emerged throughout the specialization of the different lineages. The bivalve-specific CTL subfamily A, shared by all bivalves, was of particular interest since it consisted of small secreted (signal peptide) single-domain CTLs with a specific expression pattern in *Mytilus galloprovincialis*, mainly expressed in mucosal tissues such as gills and digestive glands. The modulation of several of these proteins with different transcriptomic stimuli is in line with the importance of CTLs as recognition receptors in the mucosal tissues of bivalves [60].

Diversity related to the C-type lectin-like gene families was also observed in the domain architectures of CTLDcps. Most plant lectins are multidomain proteins that originated by modular rearrangement of protein domains during evolution [61]. Vertebrate CTLDcps are classified by their additional domains, some of which are conserved in invertebrates, indicating ancestral domain functions in a common ancestor. However, most metazoan CTLDcps are generated by species-specific domain arrangements [19,62]. Our data revealed orthologous groups of CTLDcps that conserved the same domain structure, while in other cases, clear domain rearrangements were observed in the orthologs of certain species. Groups of bivalve proteins with unique CTLDcp architectures and without orthologs outside of bivalves were also revealed. Evolutionary mechanisms such as duplication, fusion, fission, domain gain, and domain loss drive protein domain rearrangements, which are generally associated with environmental adaptation [63]. The combination of CTLs with different additional domains is implicated in the generation of new protein functions involved in defense, signaling, or development processes and in increasing the functional diversification capacity of these lectins [60,64]. The unique domain architectures found in bivalves included domains of known immune importance, such as the cell wall integrity and stress response component domain or WSC (which is a carbohydrate binding domain), sushi domains (related to complement system control), scavenger receptors (pathogen binding), apextrin or immunoglobulins, among others, indicating novel functions related to immunological specificity [65,66,67,68]. However, other domains are likely implicated in different functions that are not alien to CTLs, such as CUB, which is related to developmentally regulated proteins [69]. Bivalve CTLDcps deserve further investigation, not only those uncharacterized bivalve proteins from category “C” but also CTLDcps from categories “A” and “B”, to determine how domain rearrangements affect their function in comparison to their vertebrate orthologs.

Host-pathogen interactions have driven the evolution of immune receptors. In each species, C-type lectins evolved according to their pathogenic context, for example, with specificity toward deep-sea bacteria as in deep-sea hydrothermal vent animals such as the tubeworm *Alaysia* [70,71]. Gene families related to stress and immune response are generally expanded in bivalves as a conserved adaptation to their life strategy, providing them with a functional diversity in key genes to respond specifically to the challenges of their sessile life strategy as seawater filter feeders [24]. The expression analyses performed in the current work demonstrated distinct modulation of mussel C-type lectin-like proteins with abiotic and pathogenic stimuli, indicating the expected functional diversification in bivalve repertoires. CTLs with hemocyte expression can be related to roles as immune effectors, agglutinating pathogens and promoting phagocytosis by defensive cells [38,39]. In this sense, we detected CTLs modulated with bacterial or viral stimuli that could be implicated in their recognition or opsonization [34,72]. Several CTLs were modulated with two successive *Vibrio splendidus* infections (graphs 19–20 in Figure 5), in accordance to the specific recognition and agglutination of *Vibrio* bacteria that has been demonstrated in CTLs from different mollusks [73,74]. In bivalves, CTLs are of great importance in mucosal functions, implicated in both immune and digestive processes [60]. The fact that a bivalve-specific expanded CTL subfamily was expressed mainly in mucosal tissues points toward a conservation of these important mucosal functions in bivalve species. These mucosal CTLs presented strong modulation with different toxins in our data (graphs 14–18 in Figure 5) and would correspond to the CTLs that are normally modulated in bivalve mucosal tissues stimulated with toxin-producing organisms [75,76]. The capacity to recognize and bind different compounds and pathogens has been demonstrated for bivalve CTLs [34], and mucosal tissues, such as gills, are of key importance in the first steps of the immune response by recognizing incoming pathogens and triggering the defensive response [59]. Particle selection and recognition have been demonstrated in the binding of ligands by mucus CTLs [41]. Since mucosal tissues are the first barrier encountered by filtered particles, the conserved CTL subfamily with mucosal expression identified in this work could be related to the specific recognition of ligands in these barriers, discerning harmful particles that must be eliminated.

This work studied in detail for the first time the CTL repertoires of bivalves, the most expanded ones in all metazoans, revealing evidence of the functional specialization directing their expansions in a lineage-specific manner. Specific subfamilies with different degrees of conservation were also described, building an evolutionary history for these genes. Due to the information derived from their evolutionary information and transcriptomic modulation, specific CTL subfamilies and CTLDcps revealed in this work could be of interest for future characterization studies.

## 4. Materials and Methods

### 4.1. Screening of C-Type Lectin-like Proteins in Metazoan Genomes

Genomes from different metazoan species were downloaded, and their proteins were retrieved. Accession IDs from each genome are found in Supplementary File S1. These proteins were filtered with the agat toolkit scripts to keep only the longest isoform per gene [77]. The completeness of each genome was tested with BUSCO analyses using the metazoa_odb10 database [78].

Domains encoded in the filtered proteins of each genome were analyzed using Pfamscan and the Pfam database [79]. All proteins containing a C-type lectin-like domain (accession number PF00059.21) were retrieved. This approach was used to quantify the number of C-type lectin-like proteins in each species, differentiating between the proteins that contained only C-type lectin-like domains (CTLs) and the proteins that contained C-type lectin-like domains and other domains (CTLDcps).

### 4.2. Orthology Analyses

Orthology analyses were performed with Orthofinder [80,81] using proteins from each genome filtered for the longest isoform per gene. One orthology analysis was performed using all metazoan species indicated in Supplementary File S1, and another one was performed using species from the Mollusca phylum. From these analyses, orthology groups (orthogroups) including proteins that contained only C-type lectin-like domains (CTLs) and proteins containing a C-type lectin-like and other domains were selected. To analyze the differences and similarities in the orthology distribution of CTLs among species, a presence/absence matrix was constructed with data regarding the presence of CTLs in a particular orthogroup for each species. To visualize these data, heatmaps were made using pheatmap (version 1.0.12), and PCAs were performed using ggplot (version 3.3.6) [82].

### 4.3. Phylogenetic Analyses of Mollusk CTLs

Proteins encoded in the *Mytilus galloprovincialis* genome were filtered to select those that contained only C-type lectin-like domains (CTLs) using seqkit (version 2.3.0) [83]. Using these sequences, a multiple alignment was performed with MAFFT (version 7) [84]. AliView (version 1.28) [85] was used for alignment visualization. Afterward, PhyML [86] was used to build the phylogenetic tree. The phylogenetic analysis was performed using automatic evolutionary model selection [87]. The same analysis was performed for *Crassostrea gigas* and for the four cephalopod species analyzed in this work, *Octopus bimaculoides*, *O. sianensis*, *Sepia pharaonis*, and *Architeuthis dux*, due to the significantly low number of C-type lectin-like proteins present in cephalopods. The obtained phylogenetic trees were annotated in iTOL [88], indicating the main CTL subfamilies in each species. Heatmaps were constructed to show the conservation degree of such subfamilies, revealing which ones were shared between both mollusk classes and which were class or genus specific. The presence of signal peptides and transmembrane domains in these lectins was analyzed with Phobius [89].

### 4.4. Expression Analyses

An expression dataset constructed with 252 *M. galloprovincialis* transcriptomic samples (Supplementary File S2) mapped with salmon [90] against the “mg^3^” assembly of the mussel reference genome [23] was used to study the expression of the C-type lectin-like gene family. The expression levels in five different sample types (digestive gland, gill, hemocytes, mid-trochophore larvae, and mantle) were analyzed, as well as the modulation with the different transcriptomic stimuli included in the dataset, to search for interesting expression patterns in the *M. galloprovincialis* proteins. For the study of transcriptomic modulation, samples from the dataset belonging to specific experiments of interest (Supplementary File S3) were separated, and their counts were input into DESeq2 [91] to find differentially expressed genes. The resulting data were filtered using a *p* value threshold below 0.05.

## Data Availability

The data used in the genomic and transcriptomic analyses performed in this work are available in public databases. Accession numbers can be found in the Supplementary Materials.

## Supplementary Materials

The following Supporting Information can be downloaded at https://www.mdpi.com/article/10.3390/md21040254/s1: Supplementary File S1: Analyzed metazoan genomes; Supplementary File S2: SRA transcriptomic samples analyzed in the expression dataset; Supplementary File S3: Particular SRA transcriptomic samples belonging to specific experiments in which modulated genes were analyzed.

## References

[1] Hoffmann J.A., Kafatos F.C., Janeway C.A., Ezekowitz R.A. (1999). Phylogenetic perspectives in innate immunity. Science.

[2] Medzhitov R., Janeway C. (2000). Innate immune recognition: Mechanisms and pathways. Immunol. Rev..

[3] Rini J.M. (1995). Lectin structure. Annu. Rev. Biophys. Biomol. Struct..

[4] Goldstein I.J., Hayes C.E. (1978). The lectins: Carbohydrate-binding proteins of plants and animals. Adv. Carbohydr. Chem. Biochem..

[5] Nizet V., Varki A., Aebi M., Varki A., Cummings R.D., Esko J.D., Stanley P., Hart G.W., Aebi M., Darvill A.G., Kinoshita T., Packer N.H., Prestegard J.H. (2015). Microbial lectins: Hemagglutinins, adhesins, and toxins. Essentials of Glycobiology.

[6] Sharon N., Lis H. (2004). History of lectins: From hemagglutinins to biological recognition molecules. Glycobiology.

[7] Jiang S.-Y., Ma Z., Ramachandran S. (2010). Evolutionary history and stress regulation of the lectin superfamily in higher plants. BMC Evol. Biol..

[8] Vasta G.R., Nita-Lazar M., Giomarelli B., Ahmed H., Du S., Cammarata M., Parrinello N., Bianchet M.A., Amzel L.M. (2011). Structural and functional diversity of the lectin repertoire in teleost fish: Relevance to innate and adaptive immunity. Dev. Comp. Immunol..

[9] Lakhtin V., Lakhtin M., Alyoshkin V. (2011). Lectins of living organisms. The overview. Anaerobe.

[10] Kumar K.K., Chandra K.L.P., Sumanthi J., Reddy G.S., Shekar P.C., Reddy B.V.R. (2012). Biological role of lectins: A review. J. Orofac. Sci..

[11] Bonnardel F., Mariethoz J., Pérez S., Imberty A., Lisacek F. (2021). LectomeXplore, an Update of unilectin for the discovery of carbohydrate-binding proteins based on a new lectin classification. Nucleic Acids Res..

[12] Fujimoto Z., Tateno H., Hirabayashi J., Hirabayashi J. (2014). Lectin structures: Classification based on the 3-D structures. Lectins: Methods and Protocols.

[13] Drickamer K. (1999). C-type lectin-like domains. Curr. Opin. Struct. Biol..

[14] Abbas A.K., Lichtman A.H., Pillai S. (2021). Cellular and Molecular Immunology E-Book.

[15] Fujita T. (2002). Evolution of the lectin–complement pathway and its role in innate immunity. Nat. Rev. Immunol..

[16] Smith L.C., Azumi K., Nonaka M. (1999). Complement systems in invertebrates. The ancient alternative and lectin pathways. Immunopharmacology.

[17] Mayer S., Raulf M.-K., Lepenies B. (2017). C-type lectins: Their network and roles in pathogen recognition and immunity. Histochem. Cell Biol..

[18] Nauta A.J., Castellano G., Xu W., Woltman A.M., Borrias M.C., Daha M.R., van Kooten C., Roos A. (2004). Opsonization with C1q and mannose-binding lectin targets apoptotic cells to dendritic Cells1. J. Immunol..

[19] Zelensky A.N., Gready J.E. (2005). The C-type lectin-like domain superfamily. FEBS J..

[20] Rao X.-J., Cao X., He Y., Hu Y., Zhang X., Chen Y.-R., Blissard G., Kanost M.R., Yu X.-Q., Jiang H. (2015). Structural features, evolutionary relationships, and transcriptional regulation of C-type lectin-domain proteins in *Manduca sexta*. Insect Biochem. Mol. Biol..

[21] Wong E.S.W., Sanderson C.E., Deakin J.E., Whittington C.M., Papenfuss A.T., Belov K. (2009). Identification of natural killer cell receptor clusters in the platypus genome reveals an expansion of C-type lectin genes. Immunogenetics.

[22] Calcino A.D., Kenny N.J., Gerdol M. (2021). Single individual structural variant detection uncovers widespread hemizygosity in molluscs. Philos. Trans. R. Soc. B Biol. Sci..

[23] Gerdol M., Moreira R., Cruz F., Gómez-Garrido J., Vlasova A., Rosani U., Venier P., Naranjo-Ortiz M.A., Murgarella M., Greco S. (2020). Massive gene presence-absence variation shapes an open pan-genome in the Mediterranean mussel. Genome Biol..

[24] Regan T., Stevens L., Peñaloza C., Houston R.D., Robledo D., Bean T.P. (2021). Ancestral physical stress and later immune gene family expansions shaped bivalve mollusc evolution. Genome Biol. Evol..

[25] Suttle C.A. (2007). Marine viruses—Major players in the global ecosystem. Nat. Rev. Microbiol..

[26] Azam F., Malfatti F. (2007). Microbial structuring of marine ecosystems. Nat. Rev. Microbiol..

[27] Gerdol M., Greco S., Pallavicini A. (2019). Extensive tandem duplication events drive the expansion of the C1q-domain-containing gene family in bivalves. Mar. Drugs.

[28] Gerdol M., Venier P., Pallavicini A. (2015). The genome of the Pacific oyster *Crassostrea gigas* Brings new insights on the massive expansion of the C1q gene family in Bivalvia. Dev. Comp. Immunol..

[29] Romero A., Dios S., Poisa-Beiro L., Costa M.M., Posada D., Figueras A., Novoa B. (2011). Individual sequence variability and functional activities of fibrinogen-related proteins (FREPs) in the Mediterranean mussel (*Mytilus galloprovincialis*) Suggest ancient and complex immune recognition models in invertebrates. Dev. Comp. Immunol..

[30] Huang B., Zhang L., Li L., Tang X., Zhang G. (2015). Highly diverse fibrinogen-related proteins in the Pacific oyster *Crassostrea gigas*. Fish Shellfish Immunol..

[31] Gerdol M. (2017). Immune-related genes in gastropods and bivalves: A comparative overview. Invertebr. Surviv. J..

[32] Gourdine J.-P., Cioci G., Miguet L., Unverzagt C., Silva D.V., Varrot A., Gautier C., Smith-Ravin E.J., Imberty A. (2008). High affinity interaction between a bivalve C-type lectin and a biantennary complex-type N-glycan revealed by crystallography and microcalorimetry. J. Biol. Chem..

[33] Unno H., Itakura S., Higuchi S., Goda S., Yamaguchi K., Hatakeyama T. (2019). Novel Ca^2+^-independent carbohydrate recognition of the C-type lectins, SPL-1 and SPL-2, from the bivalve *Saxidomus purpuratus*. Protein Sci..

[34] Wang L., Huang M., Zhang H., Song L. (2011). The immune role of C-type lectins in molluscs. Invertebr. Surviv. J..

[35] Chellapackialakshmi M., Ravi C., Elumalai P., Vaseeharan B., Lakshmi S. (2022). Investigation on mollusc lectins. Aquatic Lectins.

[36] Yang J., Wang L., Zhang H., Qiu L., Wang H., Song L. (2011). C-type lectin in chlamys farreri (CfLec-1) mediating immune recognition and opsonization. PLoS ONE.

[37] Zhang H., Wang H., Wang L., Song X., Zhao J., Qiu L., Li L., Cong M., Song L. (2009). A novel C-type lectin (Cflec-3) from *Chlamys farreri* with three carbohydrate-recognition domains. Fish Shellfish Immunol..

[38] Takahashi K.G., Kuroda T., Muroga K. (2008). Purification and antibacterial characterization of a novel isoform of the manila clam lectin (MCL-4) from the plasma of the manila clam, *Ruditapes philippinarum*. Comp. Biochem. Physiol. Part B Biochem. Mol. Biol..

[39] Wang H., Song L., Li C., Zhao J., Zhang H., Ni D., Xu W. (2007). Cloning and characterization of a novel C-type lectin from Zhikong scallop *Chlamys farreri*. Mol. Immunol..

[40] Springer S.A., Moy G.W., Friend D.S., Swanson W.J., Vacquier V.D. (2004). Oyster sperm bindin is a combinatorial fucose lectin with remarkable intra-species diversity. Int. J. Dev. Biol..

[41] Espinosa E.P., Perrigault M., Ward J.E., Shumway S.E., Allam B. (2009). Lectins Associated with the feeding organs of the oyster *Crassostrea virginica* can mediate particle selection. Biol. Bull..

[42] Chikalovets I.V., Chernikov O.V., Pivkin M.V., Molchanova V.I., Litovchenko A.P., Li W., Lukyanov P.A. (2015). A lectin with antifungal activity from the mussel *Crenomytilus grayanus*. Fish Shellfish Immunol..

[43] Sivakamavalli J., Park K., Kwak I.-S., Vaseeharan B. (2021). Purification and partial characterization of carbohydrate-recognition protein C-type lectin from *Hemifusus pugilinus*. Carbohydr. Res..

[44] Li H., Zhang H., Jiang S., Wang W., Xin L., Wang H., Wang L., Song L. (2015). A single-CRD C-type lectin from oyster *Crassostrea gigas* mediates immune recognition and pathogen elimination with a potential role in the activation of complement system. Fish Shellfish Immunol..

[45] Jia Z., Zhang H., Jiang S., Wang M., Wang L., Song L. (2016). Comparative study of two single CRD C-type lectins, CgCLec-4 and CgCLec-5, from Pacific oyster *Crassostrea gigas*. Fish Shellfish Immunol..

[46] Canesi L., Grande C., Pezzati E., Balbi T., Vezzulli L., Pruzzo C. (2016). Killing of *Vibrio cholerae* and *Escherichia coli* strains carrying D-mannose-sensitive ligands by mytilus hemocytes is promoted by a multifunctional hemolymph serum protein. Microb. Ecol..

[47] Weiss I.M., Kaufmann S., Mann K., Fritz M. (2000). Purification and Characterization of perlucin and perlustrin, two new proteins from the shell of the mollusc *Haliotis laevigata*. Biochem. Biophys. Res. Commun..

[48] Chernikov O., Kuzmich A., Chikalovets I., Molchanova V., Hua K.-F. (2017). Lectin CGL from the sea mussel *Crenomytilus grayanus* induces Burkitt’s lymphoma cells death via interaction with surface glycan. Int. J. Biol. Macromol..

[49] Liao J.-H., Chien C.-T.H., Wu H.-Y., Huang K.-F., Wang I., Ho M.-R., Tu I.-F., Lee I.-M., Li W., Shih Y.-L. (2016). A multivalent marine lectin from *Crenomytilus grayanus* possesses anti-cancer activity through recognizing globotriose Gb3. J. Am. Chem. Soc..

[50] Hasan I., Sugawara S., Fujii Y., Koide Y., Terada D., Iimura N., Fujiwara T., Takahashi K.G., Kojima N., Rajia S. (2015). MytiLec, a mussel R-type lectin, interacts with surface glycan Gb3 on Burkitt’s lymphoma cells to trigger apoptosis through multiple pathways. Mar. Drugs.

[51] Hasan I., Gerdol M., Fujii Y., Rajia S., Koide Y., Yamamoto D., Kawsar S.M.A., Ozeki Y. (2016). CDNA and gene structure of MytiLec-1, a bacteriostatic R-type lectin from the Mediterranean mussel (*Mytilus galloprovincialis*). Mar. Drugs.

[52] Terada D., Voet A.R.D., Noguchi H., Kamata K., Ohki M., Addy C., Fujii Y., Yamamoto D., Ozeki Y., Tame J.R.H. (2017). Computational design of a symmetrical β-trefoil lectin with cancer cell binding activity. Sci. Rep..

[53] Pees B., Yang W., Zárate-Potes A., Schulenburg H., Dierking K. (2016). High innate immune specificity through diversified C-type lectin-like domain proteins in invertebrates. J. Innate Immun..

[54] Gundacker D., Leys S.P., Schröder H.C., Müller I.M., Müller W.E.G. (2001). Isolation and cloning of a C-type lectin from the hexactinellid sponge *Aphrocallistes vastus*: A putative aggregation factor. Glycobiology.

[55] Gardères J., Bourguet-Kondracki M.-L., Hamer B., Batel R., Schröder H.C., Müller W.E.G. (2015). Porifera lectins: Diversity, physiological roles and biotechnological potential. Mar. Drugs.

[56] Gorbushin A.M. (2019). Derivatives of the lectin complement pathway in Lophotrochozoa. Dev. Comp. Immunol..

[57] Ning J., Zhou J., Wang H., Liu Y., Ahmad F., Feng X., Fu Y., Gu X., Zhao L. (2022). Parallel evolution of C-type lectin domain gene family sizes in insect-vectored nematodes. Front. Plant Sci..

[58] Takeuchi T., Koyanagi R., Gyoja F., Kanda M., Hisata K., Fujie M., Goto H., Yamasaki S., Nagai K., Morino Y. (2016). Bivalve-specific gene expansion in the pearl oyster genome: Implications of adaptation to a sessile lifestyle. Zool. Lett..

[59] Saco A., Rey-Campos M., Rosani U., Novoa B., Figueras A. (2021). The evolution and diversity of Interleukin-17 highlight an expansion in marine invertebrates and its conserved role in mucosal immunity. Front. Immunol..

[60] Wang W., Gong C., Han Z., Lv X., Liu S., Wang L., Song L. (2019). The lectin domain containing proteins with mucosal immunity and digestive functions in oyster *Crassostrea gigas*. Fish Shellfish Immunol..

[61] Van Holle S., De Schutter K., Eggermont L., Tsaneva M., Dang L., Van Damme E.J.M. (2017). Comparative study of lectin domains in model species: New insights into evolutionary dynamics. Int. J. Mol. Sci..

[62] Wood-Charlson E.M., Weis V.M. (2009). The diversity of C-type lectins in the genome of a basal metazoan, *Nematostella vectensis*. Dev. Comp. Immunol..

[63] Moore A.D., Bornberg-Bauer E. (2012). The dynamics and evolutionary potential of domain loss and emergence. Mol. Biol. Evol..

[64] Bashton M., Chothia C. (2007). The generation of new protein functions by the combination of domains. Structure.

[65] Gerdol M., Luo Y.-J., Satoh N., Pallavicini A. (2018). Genetic and molecular basis of the immune system in the brachiopod *Lingula anatina*. Dev. Comp. Immunol..

[66] Freeman M., Ashkenas J., Rees D.J., Kingsley D.M., Copeland N.G., Jenkins N.A., Krieger M. (1990). An ancient, highly conserved family of cysteine-rich protein domains revealed by cloning type I and type II murine macrophage scavenger receptors. Proc. Natl. Acad. Sci. USA.

[67] Reid K.B.M., Day A.J. (1989). Structure-function relationships of the complement components. Immunol. Today.

[68] Huang G., Huang S., Yan X., Yang P., Li J., Xu W., Zhang L., Wang R., Yu Y., Yuan S. (2014). Two apextrin-like proteins mediate extracellular and intracellular bacterial recognition in Amphioxus. Proc. Natl. Acad. Sci. USA.

[69] Bork P., Beckmann G. (1993). The CUB domain: A widespread module in developmentally regulated proteins. J. Mol. Biol..

[70] Van den Berg L.M., Gringhuis S.I., Geijtenbeek T.B.H. (2012). An evolutionary perspective on C-type lectins in infection and immunity. Ann. N. Y. Acad. Sci..

[71] Jin Q., Sun Q., Zhang J., Sun L. (2018). First characterization of two C-type lectins of the *Tubeworm alaysia* sp. from a deep-sea hydrothermal vent. Dev. Comp. Immunol..

[72] Huang M., Song X., Zhao J., Mu C., Wang L., Zhang H., Zhou Z., Liu X., Song L. (2013). A C-type lectin (AiCTL-3) from bay scallop *Argopecten irradians* with mannose/galactose binding ability to bind various bacteria. Gene.

[73] Chen H., Cai X., Qiu H., Fang J., Wu X. (2021). A novel C-type lectin from *Crassostrea gigas* involved in the innate defense against *Vibrio alginolyticus*. Biochem. Biophys. Res. Commun..

[74] Wang N., Whang I., Lee J. (2008). A novel C-type lectin from abalone, *Haliotis discus discus*, agglutinates *Vibrio alginolyticus*. Dev. Comp. Immunol..

[75] Dou M., Jiao Y., Zheng J., Zhang G., Li H., Liu J., Yang W. (2020). De novo transcriptome analysis of the mussel *Perna viridis* after exposure to the toxic dinoflagellate *Prorocentrum lima*. Ecotoxicol. Environ. Saf..

[76] Gerdol M., De Moro G., Manfrin C., Milandri A., Riccardi E., Beran A., Venier P., Pallavicini A. (2014). RNA sequencing and de novo assembly of the digestive gland transcriptome in *Mytilus galloprovincialis* fed with toxinogenic and non-toxic strains of *Alexandrium minutum*. BMC Res. Notes.

[77] Dainat J., Hereñú D., Pucholt P. (2020). AGAT: Another Gff Analysis Toolkit to handle annotations in any GTF. Zenodo.

[78] Manni M., Berkeley M.R., Seppey M., Simão F.A., Zdobnov E.M. (2021). BUSCO update: Novel and streamlined workflows along with broader and deeper phylogenetic coverage for scoring of eukaryotic, prokaryotic, and viral genomes. Mol. Biol. Evol..

[79] Finn R.D., Bateman A., Clements J., Coggill P., Eberhardt R.Y., Eddy S.R., Heger A., Hetherington K., Holm L., Mistry J. (2014). Pfam: The protein families database. Nucleic Acids Res..

[80] Emms D.M., Kelly S. (2015). OrthoFinder: Solving fundamental biases in whole genome comparisons dramatically improves orthogroup inference accuracy. Genome Biol..

[81] Emms D.M., Kelly S. (2019). OrthoFinder: Phylogenetic orthology inference for comparative genomics. Genome Biol..

[82] Wickham H. (2016). Ggplot2: Elegant Graphics for Data Analysis.

[83] Shen W., Le S., Li Y., Hu F. (2016). SeqKit: A cross-platform and ultrafast toolkit for FASTA/Q File manipulation. PLoS ONE.

[84] Katoh K., Rozewicki J., Yamada K.D. (2019). MAFFT online service: Multiple sequence alignment, interactive sequence choice and visualization. Brief. Bioinform..

[85] Larsson A. (2014). AliView: A fast and lightweight alignment viewer and editor for large datasets. Bioinformatics.

[86] Guindon S., Gascuel O. (2003). A simple, fast, and accurate algorithm to estimate large phylogenies by maximum likelihood. Syst. Biol..

[87] Lefort V., Longueville J.-E., Gascuel O. (2017). SMS: Smart model selection in PhyML. Mol. Biol. Evol..

[88] Letunic I., Bork P. (2007). Interactive tree of life (ITOL): An online tool for phylogenetic tree display and annotation. Bioinformatics.

[89] Käll L., Krogh A., Sonnhammer E.L.L. (2007). Advantages of combined transmembrane topology and signal peptide prediction—The Phobius web server. Nucleic Acids Res..

[90] Patro R., Duggal G., Love M.I., Irizarry R.A., Kingsford C. (2017). Salmon provides fast and bias-aware quantification of transcript expression. Nat. Methods.

[91] Love M.I., Huber W., Anders S. (2014). Moderated estimation of fold change and dispersion for RNA-seq data with DESeq2. Genome Biol..

